# Development and Validation of a Deep Learning Based Automated Minirhizotron Image Analysis Pipeline

**DOI:** 10.34133/2022/9758532

**Published:** 2022-05-28

**Authors:** Felix Maximilian Bauer, Lena Lärm, Shehan Morandage, Guillaume Lobet, Jan Vanderborght, Harry Vereecken, Andrea Schnepf

**Affiliations:** ^1^Institute of Bio-and Geosciences, Agrosphere (IBG-3), Forschungszentrum Jülich GmbH, 52425 Jülich, Germany; ^2^Institute of Soil Science and Land Evaluation, University of Hohenheim, 70559 78 Stuttgart, Germany

## Abstract

Root systems of crops play a significant role in agroecosystems. The root system is essential for water and nutrient uptake, plant stability, symbiosis with microbes, and a good soil structure. Minirhizotrons have shown to be effective to noninvasively investigate the root system. Root traits, like root length, can therefore be obtained throughout the crop growing season. Analyzing datasets from minirhizotrons using common manual annotation methods, with conventional software tools, is time-consuming and labor-intensive. Therefore, an objective method for high-throughput image analysis that provides data for field root phenotyping is necessary. In this study, we developed a pipeline combining state-of-the-art software tools, using deep neural networks and automated feature extraction. This pipeline consists of two major components and was applied to large root image datasets from minirhizotrons. First, a segmentation by a neural network model, trained with a small image sample, is performed. Training and segmentation are done using “RootPainter.” Then, an automated feature extraction from the segments is carried out by “RhizoVision Explorer.” To validate the results of our automated analysis pipeline, a comparison of root length between manually annotated and automatically processed data was realized with more than 36,500 images. Mainly the results show a high correlation (*r* = 0.9) between manually and automatically determined root lengths. With respect to the processing time, our new pipeline outperforms manual annotation by 98.1-99.6%. Our pipeline, combining state-of-the-art software tools, significantly reduces the processing time for minirhizotron images. Thus, image analysis is no longer the bottle-neck in high-throughput phenotyping approaches.

## 1. Introduction

Roots are an essential component of the global biosphere. They are mainly responsible for the acquisition of the resources water and nutrients for the entire plant. In most ecosystems, these resources are the limiting factors for growth of plant organs and yield [1]. Water and nutrient uptake are directly linked to the parameters defining the root system, like length, diameter, or branching. Therefore, collecting information about the root system becomes increasingly significant. In order to improve water and nutrient uptake of plants for specific soil and climatic conditions, it is essential to obtain information about the root system architecture of plant species that have been shown to be beneficial for the given conditions [2]. For plant breeding, this will help to develop new genotypes which are able to cope better with, e.g., drought-stress and are more efficient in nutrient uptake [3]. This will not only help to increase the cultivated area for certain species, but it might also lead to higher yields. Especially this applies to locations with less suitable environments for a highly productive agriculture. The negative impact on the soil should be minimized at the same time [4].

The direct observation of roots is difficult, because the root system is surrounded by soil, making it challenging to visually measure the roots. To avoid that measurements heavily disturb the plant and its environment, permanent installed equipment, like rhizotubes, or the construction of a minirhizotron is crucial [5]. Minirhizotrons are useful tools to collect data about the root system without disturbing the environment of the roots or the plant itself. Moreover, they allow root observations over the whole vegetation period at a high temporal resolution and the comparison of different vegetation periods and crop types. Transparent rhizotubes, installed below ground, function as a window in the soil. Guided scanners and camera systems provide high-resolution images of the roots and the surrounding soil. Consequently, the noninvasive root measurements can be repeated multiple times during the growing period under in situ conditions. However, large minirhizotron facilities include tubes in different depth levels. Measurements in several depths and time-lapse observations result in big datasets that often consist out of 10,000 images and more [6]. Images provided by minirhizotrons strongly differ from, e.g., root scans gained from excavated and washed roots [7]. Various soil conditions around the tubes in different depths lead to a wide range of heterogeneous images with different characteristics. Beside the actual roots, soil structures and disturbing fragments, including small animals, are depicted. Different soil conditions in various depths and at varying locations lead to varying color and light conditions and therefore make the automated processing of minirhizotron images a challenging task [8].

To analyze roots, mainly two steps are needed, the segmentation of root objects and the object quantification [9]. Due to the heterogeneity within minirhizotron images, the segmentation is very complicated. Different analysis approaches emerged, represented by a numerous collection of software tools, and designed to extract the information about the root system [10]. These tools work manually or in a (semi-)automated way. Manual annotation tools for minirhizotron images, like “WinRhizoTRON” (Regent Instruments Incl.) or RhizoTrak [11], rely on the human interaction with each individual image taken, to track each root by hand. It requires the user to follow every root depicted in the image by hand and mark start, branch, and endpoints. Semiautomated and automated approaches with software tools exist to facilitate and speed up the postprocessing of the images [8]. Filter algorithms used to increase the contrast between root and background and to find root structures by typical geometrical shapes were proposed by several authors [7, 12, 13]. Semiautomated software like “RootSnap!” (CID Bioscience) and “Rootfly” [14] require a manual annotation, but also provide root suggestions by a filter created on an initial dataset. Consequently, most of these programs are strictly limited to certain type of images, like high-contrast root scans [15]. Eventually, this has the consequence that the annotation of the roots in most minirhizotron images needs to be done almost exclusively manually. Depending on the number of images taken and the number and length of roots, the manual and semiautomated analysis can take weeks to years. Previous studies found that the estimated amount of minirhizotron images, annotated with an annotation software, was between 17 and 38 images h ^−1^ [16]. Adapted to the working routine with “Rootfly,” it takes 1-1.5 h annotation time for an image area of 100 cm^2^ depicted soil [17]. Further, the results underlie the subjectivity of the annotator, because annotations are done according to personal experiences and knowledge of the annotator.

Deep learning has developed to the gold standard of machine learning methods within the recent years. Deep neural networks are able to learn from big datasets and provide outstanding results on complex cognitive challenges, even beating human performance in some application fields [18]. Convolutional Neural Networks (CNN), a subclass of deep learning models, have been created to deal with data in the shape of multiple arrays and are therefore suitable for high-dimensional data like images [19]. They have the potential to perform a decent automated detection of regions of interests within a heterogeneous and noisy dataset [20]. Transferred to the analysis of minirhizotron images, CNNs should have the capability to precisely identify and segment roots in images, where the roots cannot be segmented sufficiently by, e.g., explicitly programmed thresholds or filter algorithms. CNNs were already used successfully to localize plant organs, including roots [21–24]. However, the use of CNNs has mainly been proven on data originating from controlled environment, like lab experiments [15]. Furthermore, they are often limited to the use of one or a few fixed pretrained neural network models [25], or they are not easily usable for non-IT professionals [26]. The main reason for this is the required knowledge and competences in machine learning and programming needed to create a CNN-based system. Especially the data partition between training and validation, the process of annotation and the setup of network architecture make the use of CNNs complicated [27]. Although the use of CNNs is promising for root segmentation and the first approaches to use CNNs to segment roots have been successfully accomplished with, e.g., the “SegRoot” networks, it is not subject of many published studies and not yet widely used as phenotyping tool for root traits [28]. To make the advantages of CNNs widely utilizable, a software, combining the annotation, training, and segmentation process with CNN together in an interface easy to handle is the key for general use of neural networks for automated root segmentation. The recently published software tool “RootPainter” is one of the most promising approaches for this task [29].

However, fast and reliable segmentation is only the first step of root analysis. For the root quantification, another tool is required to obtain morphological and topological features from segmented images. For this task conventional automated root analysis tools, “WinRhizo” (Regent Instruments Incl.) and “IJ_Rhizo” [30] can be used. Recent progress in the development of root system feature extraction from high-contrast images or scans has resulted in new software tool with the ability of extracting multiple features with a high precision. On the front line of current developments is the new software “RhizoVision Explorer,” providing the functions to accurately skeletonize a high-contrast segmented image, to correct the skeleton and deriving several features from it [31].

The aim of our study is to develop a generally applicable, automated analysis pipeline, based on state-of-the-art technologies and software to extract root traits from minirhizotron images. This includes data annotation for neural network training, segmentation, and feature extraction. The automated analysis pipeline has to meet the requirements in (i) availability and feasibility, (ii) accuracy and comparability, and (iii) speed and efficiency. It was an important requirement to us that this workflow should be feasible for root scientists, who only have basic knowledge in programming or computer science. This workflow should make fast root phenotyping easily accessible for newcomers in root science and lower the time and effort needed to get into the topic. Therefore, it relies on already published software. This workflow further should underline the practicability of deep learning phenotyping tools for the scientific root analysis routine. All software required to use this automated root image analysis pipeline is freely available and easy to operate. Another key advantage of our study is the scope of data used for validation and comparison and the concomitant claim to a general validity of this pipeline. To test and validate the automated analysis pipeline, datasets obtained from several years and two minirhizotron facilities were processed and compared to previously manual annotated data [6, 32–34]. Previous studies evaluating the results of a CNN-automated image analysis for root images originating from (mini)rhizotrons used between 40 and 857 images [17, 25, 28]. In our test, we evaluated the results of more than 107,000 images of which we used more than 36,500 for a direct one-to-one comparison of manual human annotation to our automated analysis pipeline. The images represent different in situ conditions. In this paper we will present the detailed procedure on operating the automated analysis pipeline and compare its performance to a previously done manual annotation for a decent evaluation.

## 2. Materials and Methods

### 2.1. Experimental Test Site

The data used for the automated analysis pipeline were collected at the two minirhizotron facilities at the Selhausen test site of the Forschungszentrum Julich GmbH (50° 52′ 07.8″ N, 6° 26′ 59.7″ E), Germany [35, 36]. The field, in which the minirhizotron facilities are located, has a slight incline with a slope of under 4°. The two minirhizotron-facilities are approximately 150 m apart. The minirhizotron facility located at the top of the field is hereafter referred to as RUT (rhizotron upper terrace) and the minirhizotron at the lower part of the field as RLT (rhizotron lower terrace). The thickness of the soil layer with silty loam texture varies strongly along the field slope. While it is not present at the top, its thickness at the bottom is up to 3 m. At RUT, the gravel content is 60%, while at RLT, it is only 4%. Both facilities contain 54 horizontally installed, transparent tubes with each a length of 7 m and an outer diameter of 6.4 cm. The tubes are separated into three plots with each three vertical, slightly shifted (10 cm) rows of six tubes, where three different treatments can be studied. The tubes in each row are installed in -10 cm, -20 cm, -40 cm, -60 cm, -80 cm, and -120 cm depth. Past treatments include different irrigation patterns (sheltered, rainfed, irrigated), different sowing densities and dates (later sowing in sheltered plot), or cultivar mixtures (two single cultivar treatments and one mixture). The two minirhizotron facilities were installed in 2012 (RUT) and 2014 (RLT), respectively. Further construction details are explained in [6].

### 2.2. Data Acquisition

Two different camera systems manufactured by Bartz (Bartz Technology Corporation) and VSI (Vienna Scientific Instruments GmbH) were used to capture the root images in the minirhizotrons. Both camera systems are designed to be used manually. A regular measurement produces 40 images per tube. 20 images are taken 80° clockwise and 20 images 80° counter-clockwise from the tubes top point [6, 32, 37, 38]. In this study, the collected images of two crop growing seasons from 2015/2016 to 2017 were taken into account. Depending on the year and measurement date, either the Bartz or the VSI system was used. The crops cultivated at the test site and used for this study were *Triticum aestivum* cv. Ambello in 2015/2016 (winter wheat) and in 2017 *Zea mays* cv. Zoey.  Table 1 gives an overview on camera system used, the resolution of the images, measurement years, measured time period and cultivars observed. Depending on crop growing season, the total amount of measurement dates varied between 21 and 38. The amount of images, taken at one measurement date, varied according to the amount of tubes measured at this measurement date (Table S1). This was depending on the state of vegetation evaluated in field.

Over the past years, the root images collected in the minirhizotron facilities in Selhausen were analyzed manually, using “Rootfly” as a semiautomated tracking tool for the root length and root counts [6, 14, 32, 33, 38]. In this study, the images of the years 2015/2016 and 2017 were analyzed. The manual annotation of 2015/2016 and 2017 has been already published in [32, 34]. Further a subsample of the root images was manually annotated by two persons separately in “Rootfly.” 1,760 images were used for the comparison between both annotators, and the annotators and the results of the automated analysis pipeline, to test if there are differences in terms of human subjectivity.

### 2.3. Software Tools

Our proposed automated minirhizotron image analysis pipeline is based on two software tools for the segmentation [29] and the automated feature extraction [31]. Furthermore, scripts to convert the segmented images and to analyze the outcome are available. For an easy accessibility, all scripts are available together within the GUI of the executable “RootAnalysisAssistance” (Supplementary Material). The conversion of the segmented images is also possible within “RootPainter.”

#### 2.3.1. Segmentation

“RootPainter,” a software tool for the deep learning segmentation of biological images with an included annotation function, provides an interactive training method within a GUI, using a U-net-based CNN. U-net was developed to train with less images for a more precise segmentation and is therefore suitable when it comes to images where the manual annotation is especially time- and labor-consuming [17, 39]. “RootPainter” was developed to make training-data creation, annotation, and network-training accessible for ordinary users. It provides a dataset creation function, which allows an easy selection of training images and cropping them in multiple tiles and to a suitable size for the interactive training. The training mode provides an interactive graphical platform to manually annotate a small part of the dataset and create a neural network model. Further, a mode to segment whole image directories at once is provided. For training and segmentation, a graphics processing unit (GPU) is required [29]. However, a full minirhizotron image analysis is based on two main components: the segmentation and the root trait extraction. Although “RootPainter” provides an inbuilt function for basic root trait extraction based on the previous segmented images, it does not provide, e.g., a skeleton correction function and a comprehensive feature extraction including multiple root traits. For our pipeline, the feature extraction part should provide multiple morphological and architectural root features with a high accuracy. Furthermore, the possibility of a systematic correction function should be implied. Therefore, a platform fulfilling these requirements was used for feature extraction.

#### 2.3.2. Feature Extraction

“RhizoVision Explorer” represents the current state-of-the-art technology with a sophisticated automated root trait extraction from segmented root images, by combining the abilities of several existing root image analysis platforms. This includes skeletonization of the segments, filter, filling, smoothing, and pruning functions [31, 40]. However, like most programs for automated root system analysis, it is built for the use with binary images or high-contrast scans and therefore not suitable for minirhizotron images. The capability of “RhizoVision Explorer” is nevertheless useful when applied to already segmented minirhizotron images.

### 2.4. Analysis Pipeline

**Figure 1:**
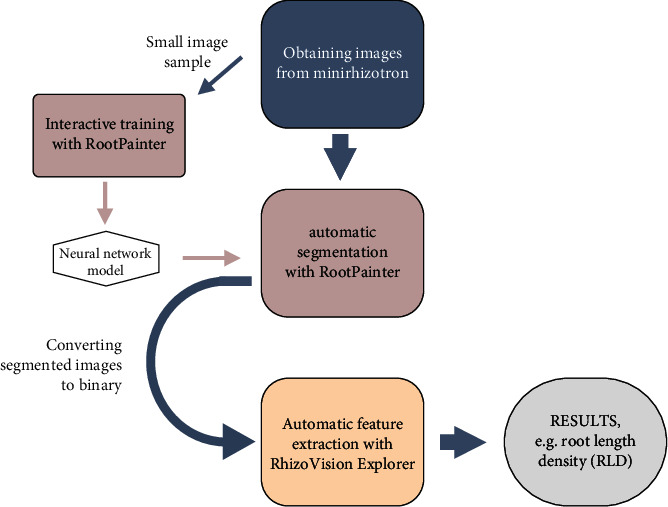
Schematic overview of the workflow of the automated analysis pipeline starting with image acquisition in the minirhizotron facility.

The starting point for the automated analysis pipeline is a directory containing the raw images captured at the minirhizotron facility. The pipeline was run on a GPU-server with 4 Nvidia GeForce RTX 2080 Ti (NVIDIA Corporation). As client, a computer with an Intel i5-8265U processor and 24 GB RAM, operated on Windows 10, was used. However, it is also possible to run the pipeline on one machine, if there is a GPU with CUDA available, or to use the “Google Colaboratory” (Google Colab). An overview of all following steps is explained in Figure 1.

#### 2.4.1. Preprocessing

The first step of the pipeline is the preprocessing of the images. Depending on the image acquisition system either an up- or down-scaling, a distortion correction is performed (supplementary data). In the same step a labeling, sorting and registration of the images are done automatically. If the images are already ready to use, this step can be omitted.

#### 2.4.2. Training

This step is only needed if no suitable neural network model exists for the targeted dataset. The process, to train a model for root segmentation, starts with the creation of a training dataset and subsequently a new project in “RootPainter.” We highly recommend to balance the training data according to the factors influencing image visually, in order to maximize the heterogeneity in the training data. Images with different quantities of roots and various root types at different locations should be included. In our case, the training dataset for one model contains a balanced amount of images from two different minirhizotron facilities, respectively, soil types, depths, tubes, and dates. We used only a small amount of images from all available images. For each camera system, a separate model was trained, because the images of the two cameras differ significantly. The annotation can be done in the GUI. The roots are annotated as “foreground” and soil and other not root-belonging fragments as “background.” After the training is started, “RootPainter” automatically creates neural network models, depending on the annotation done previously. The progress can be seen in real time, because “RootPainter” provides previews of the segmentation done by the actual model. These proposals can be corrected and supplemented by the user. The training procedure used in this study is the “corrective training.” It is intended for large datasets and therefore suitable for the minirhizotron image data. Essentially, this training approach starts with annotating a few images in detail and then continues with correcting only the false-positive and false-negative suggestions of the current model. After finishing the interactive annotation, the training is completed automatically. Further details and instructions are explained in [29].

#### 2.4.3. Segmentation

**Figure 2:**
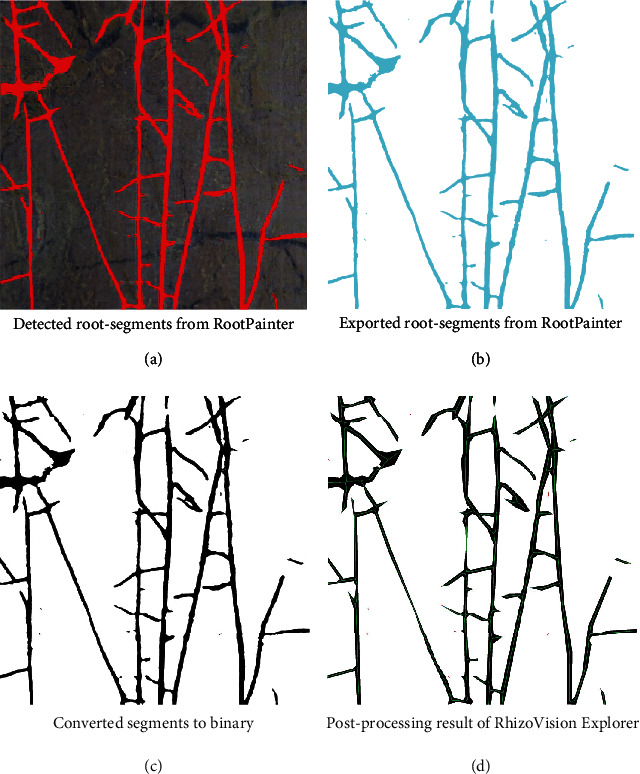
Example for one image processed by the automated root analysis pipeline. (a) The roots are “detected” by RootPainter according to the previous trained model. (b) The segmented image is exported and (c) converted to binary. (d) The last step is the skeletonization and feature extraction with RhizoVision Explorer.

The fully automated segmentation is done with the best model previously trained with a small selection of images from the corresponding measurements. To perform the fully automatic segmentation, all images have to be located in one directory. The segmentation process itself is started from the “RootPainter” main menu. For each minirhizotron image stored in the directory, one segmented image will be created (Figures 2(a) and 2(b)).

#### 2.4.4. Converting

To import the segmented images into “RhizoVision Explorer” in the next step, it is essential to convert the images to binary; otherwise, the images are not loaded properly (Figure 2(c)). This step is performed by a conversion script, which converts the monocolored segmented images to black and white images and reduces the images information to binary by only giving information for either black or white pixels. The conversion script is available as Python script or within the *RootAnalysisAssistance*-GUI. It is possible to either browse the image folders to convert manually or to process the conversion of a certain image directory in a batch mode. This option is suitable for fast processing a large amount of segmented images. The conversion option is also available within the “RootPainter”-GUI.

#### 2.4.5. Feature Extraction

The final step is the feature extraction, performed by “RhizoVision Explorer.” This is also done in batch mode. The threshold of the nonroot filter, hole filling, edge smoothing, and pruning was chosen in a standardized way and uniform for each parameter, depending on the resolution of the image. For the images resulting from the Bartz system, the threshold is 13 px and for the VSI system 20 px. This results in filtering parts smaller than 0.2 mm^2^ and filling holes bigger than 0.2 mm^2^. To minimize the influence of segmentation mistakes at the border between root and soil and thus reduce the false detection of nonexistent laterals, the minimum size for a lateral root to be detected as a branching root is the parent roots radius multiplied with 0.2 mm. The architectural and morphological information are exported as CSV, and the processed segmented images with the calculated skeleton are saved as PNG (Figure 2(d)). The feature extraction is started from the “RhizoVision Explorer” GUI. Further details and background information are explained in [40].

#### 2.4.6. Root Analysis

As the last step in addition to the feature extraction, the two-dimensional root length density (RLD) is calculated from the total root length and the window size of the image in the unit of cm cm ^−2^. Furthermore, the number of root tips and branch points, the total root length, the branching frequency, the network and surface area, the diameter (average, median, and maximal), the perimeter, and the volume can be extracted from the “RhizoVision Explorer” output CSV and applied to spatiotemporal analysis of the root system (Figure S1 and supplementary data).

### 2.5. Statistics, Data Processing, and Visualization

Python 3.8 with *Pandas 1.0.5*, *Numpy 1.18.5*, *Matplotlib 3.2.2*, *Pillow 8.2.0*, and *SciPy 1.5.0* has been used for statistics, data processing, and visualization.

The *F*_1_ score (Equation (1)) is a measure commonly used to evaluate neural network models [29]. The *F*_1_ combines precision and recall and has been designed to work on imbalanced data. Precision evaluates the percentage of all correct positive predictions, and recall indicates how many positive of all positives the model found. *F*_1_ values are bounded between 0 and 1, and the highest value is indicating perfect precision and recall.

(1) $$\begin{array}{c} F_{1}=2*\frac{\text{precision}*\text{recall}}{\text{precision}+\text{recall}}, \end{array}$$

(2) $$\begin{array}{c} \begin{array}{c} \text{precision}=\frac{{\text{T}}_{\text{P}}}{{\text{T}}_{\text{P}}+{\text{F}}_{\text{P}}} \end{array}, \end{array}$$

(3) $$\begin{array}{c} \begin{array}{c} \text{recall}=\frac{{\text{T}}_{\text{P}}}{{\text{T}}_{\text{P}}+{\text{F}}_{\text{N}}} \end{array}, \end{array}$$

where *T*_*P*_ are the true-positive, *F*_*P*_ the false-positive, and *F*_*N*_ are the false-negative pixels. The *F*_1_ score was calculated during the interactive training. True-positive pixels are correct recognized pixels, where the roots are correctly classified as roots. False-positive pixels are pixels classified as root, not including a part of a root, and false-negative pixels are pixels including parts of a root, but are classified as background.

The outcome of the automated root annotation was compared to the manual annotation by means of the Pearson correlation coefficient, both on the dataset as a whole as well as on individual measurement dates for the seasons 2017. For the same season, we calculated the mean of the total root length per image for each measurement date and used a Welch two-sample *t* test to assess whether the differences between automated analysis and manual annotation of the total root length (ΔRL) were statistically significant. Furthermore, the normalized root mean squared error (NRMSE) is calculated according to

(4) $$\begin{array}{c} \begin{array}{c} \text{NRMSE}=\frac{\sqrt{\sum_{i=1}^{n}{\left(y_{i}-\hat{y}\right)}^{2}/n}}{y_{\max}-y_{\min}} \end{array}, \end{array}$$

where *n* is the sample size, *y*_*i*_ is the *i*^*th*^ observation of *y*, and $\hat{y}$ is the predicted y value.

Additionally a linear model II regression (ordinary least products) was performed to test for fixed and proportional bias with the total root length of 2017 data. We choose this type of regression because the *x*-values might also be subject to errors [41, 42]. For each measurement date and facility, a model was fitted and the 95% confidence interval (95% CI) of slope, and intercept was calculated. We considered a fixed bias if the 95% CI of the intercept did not include 0 and there was a proportional bias if the 95% CI of the slope did not include 1.0.

The manual per image annotation with “Rootfly” of 2015/2016 data is no longer available. However, the images and mean RLD values per tube are available and therefore were used for comparison. Based on this, the RLD resulting from automated and manual analysis methods was calculated for every minirhizotron tube and measurement date (Figure S1) and compared as a proxy for a common root measurement parameter [43]. In this analysis, all growing periods 2015/2016 and 2017 were included.

## 3. Results

### 3.1. Neural Network Model Validation

The *F*_1_ for both neural network models trained for each camera system is high. The *F*_1_ for the Bartz system is 0.78 and 0.81 for VSI system model. After 60 epochs without any improvement, the neural network training was stopped automatically.

### 3.2. Comparison of Automated and Manual Annotation

Considering all images used for comparison, the overall correlation of total root length between manual annotation and automated analysis pipeline is very high with *r* = 0.9.

**Figure 3:**
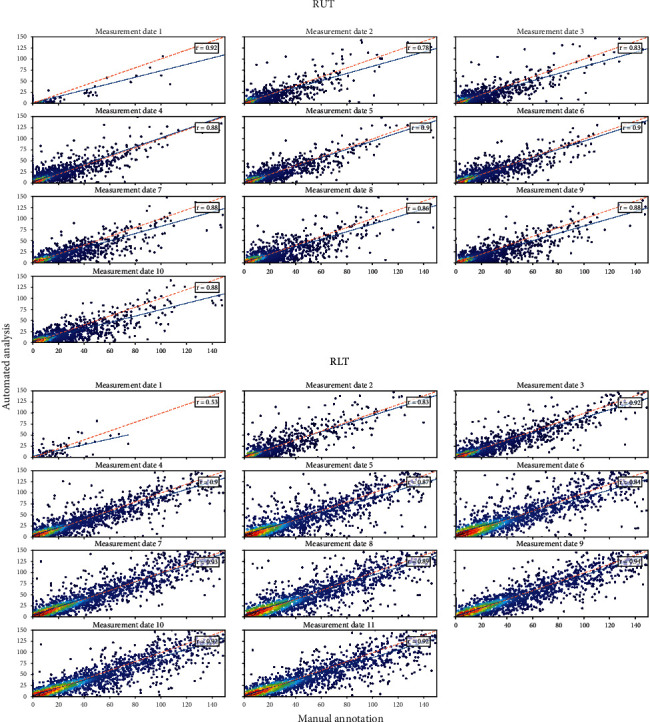
Correlation of automated and manual analyzed root length, obtained from 2017. Each measurement date is considered separately for RUT and RLT. The color represents the density.

The correlation was performed with 16,599 images taken at RUT and 21,082 images taken at RLT. For the data obtained in the growing period 2017, the correlation is high to very high (*r* = 0.77 − 0.94) for every measurement date except the first measurement date at RLT (*r* = 0.57) (Figure 3). Generally, the correlation shows an increasing trend towards later measurement dates (Table 2). *Δ*RL and NRMSE indicate low values for most measurement dates at both facilities. Regarding especially the *Δ*RL, it can be seen that the differences in the mean between the manual annotation and automated analysis pipeline in 2017 are very low (-0.5 mm (RUT) and -0.77 mm (RLT)). However, the *t* test indicates that there are no significant differences between the mean of total root length except for measurement date 4 at RUT. The slope of the linear regression models is slightly under one in most cases and the intercept marginally higher than 0 for all measurement dates. Both fixed and proportional bias were detected within almost every measurement date (Table S2).

**Figure 4:**
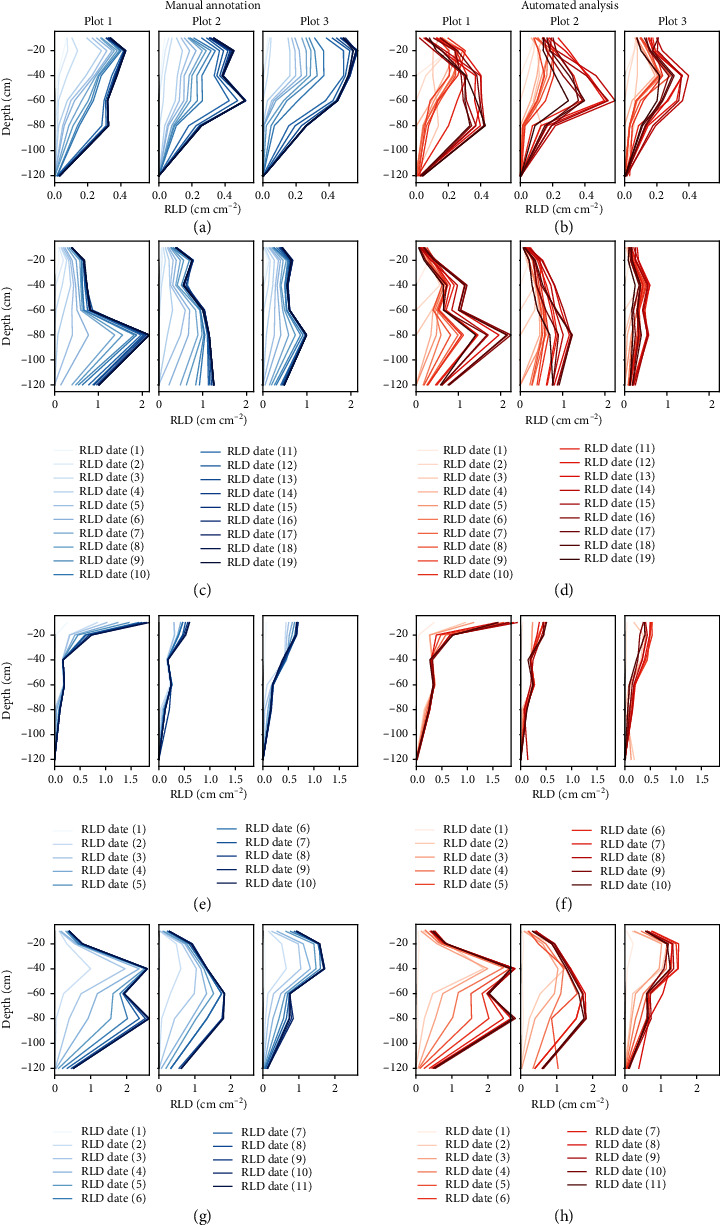
Comparison of RLD of the date obtained from images originating from two minirhizotrons in the growing season 2015/2016 and 2017, separated by plots grown with different treatments. The images were analyzed by hand (blue: manual) and by the automated analysis pipeline (red: automated). 2015/2016: (a) RUT manual, (b) RUT automated, (c) RLT manual, and (d) RLT automated. 2017: (e) RUT manual, (f) RUT automated, (g) RLT manual, and (h) RLT automated.

Regarding the RLD values from 2015/2016, one specific difference between manual and automated analysis is visible. Until the 14th measurement date, the RLD is continuously increasing and then stagnating in the 2015/2016 data resulting from manual annotation. The RLD from the automated analysis follows the same trend but decreases from 14th measurement date continuously. Beyond this, the RLD curves of both methods are very consistent (Figure 4). In 2017 datasets, only negligible differences between manual and automated analysis method are recognizable, except for the first measurement date at RLT (Figure 4(f), Figure S2b) and first two dates and a small peak at the fourth measurement at RUT (Figure 4(h)).

The comparison between two human annotators and each annotator and the automated analysis pipeline separately shows that the correlation between the person 1 and the pipeline is *r* = 0.92 and the correlation between person 2 and the pipeline is *r* = 0.79. The correlation between both persons is the lowest (*r* = 0.73).

### 3.3. Time Evaluation

**Figure 5:**
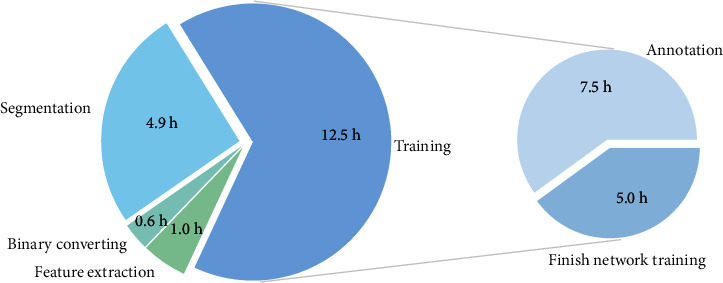
Time requirements to run the automated analysis pipeline for a sample of 25,000 images. (a) All subprocesses together. (b) Share of the neural network training, which is only required when no suitable model is available.

The time required to train the neural network model mostly depends on the amount of images included in the training dataset. Approximately 65% of the time needed is used for training of the deep neural network. The annotation takes 40% of the time, based on a mean of 200 annotated images h ^−1^. The range it took to annotate one image was between 1 and 180 s per image, depending on the accuracy of the proposed segmentation. The time required for annotation decreases significantly with increasing training time. The mean time needed by the network for the training of a dataset of 1,500 images was approximately 5 h, excluding the real-time training during the annotation. This is approximately 25% of the entire processing time. Segmentation took around 27% of the total time. With 4 Nvidia GeForce RTX 2080 Ti GPUs and a batch size of 12, the segmentation took around 0.7 s per image. Converting the segmented to binary images and the final feature extraction took around 8% of the time (Figure 5).

## 4. Discussion

### 4.1. Availability and Feasibility

The availability is the parameter for how easily accessible all components of the automated pipeline are for everyone. The feasibility defines how easy the proposed pipeline and with that the required software can be operated. The equipment needed to apply the new workflow requires a computer with a powerful GPU, or alternatively a basic computer, an additional server with powerful GPUs, and a network-connection between both. Furthermore, the software packages of “RootPainter” and “RhizoVision Explorer” are needed, and the conversion and analysis script are required. All this is open-source available [17, 31]. All software can be found in the “Data Availability.”

The training of the model requires interaction with “RootPainter,” if the user wants to start the training of a new model or corrects a segmentation within the training process. This step is therefore not fully automated. All other components of the automated analysis pipeline are automated. The interactive mode of the training represents a major time saving compared to the conventional separation of the training step and the application step. Adaptations to the model can be done “on the fly” with little time investment, facilitating, e.g., the adaptation to new types of images. Once the model is trained, the human interaction needed to apply the pipeline is reduced to a few “clicks.” With a suitable model available, the user has to interact actively three times with the automated pipeline, (1) to start the segmentation, (2) to convert the segments to binary, and (3) to start the feature extraction. No deeper knowledge in computer science is needed, because all intermediate steps are available within a GUI. However, the first implementation of the “RootPainter” environment at the server part of the setup requires basic knowledge in server administration or support.

In contrast to manual or semiautomated operated root analysis programs, like different tools based on “ImageJ,” “DART,” “GiA Roots,” “SmartRoot,” “EZ-Rhizo,” or “Rootfly,” the expenses in time, knowledge, and experiences required to apply the automated workflow are much lower. This is granted due to the very small interactions needed for the automated analysis pipeline [30, 44–47].

### 4.2. Accuracy and Comparability

The accuracy evaluates the automated analysis pipeline in terms of reliability and exactness of the generated data. Comparability is given, if the results of the automated analysis pipeline can be compared to the outcome of previously evaluated data of the same kind, like the manual annotation performed with “Rootfly.” The most important characteristic of the automation of plant data analysis is the reliability of the generated datasets. Therefore, the accuracy of the observed root traits has to be as close to the ground truth as possible [5]. In our study, we used the manual annotation of the roots as comparison. The manual annotation was performed by different persons and over a long time period. Consequently, a certain subjectivity was included in this process.

Generally, the results for 2017 data analyzed automatically and manually are very close to each other, indicating a general great fit of the models used for images originating from 2017.

However, there is a fixed and proportional bias between automated analysis and manual annotation, showing a minor but systematic underestimation of total root length from the automated analysis (Table S2) that increases slightly to the later measurement dates (see also the negative ΔRL values in Table 1). This originates from the fact that the neural network model is only able to segment roots, if they are also visible by the human eye. Rarely, small parts of roots are covered by soil, and this can only be compensated to a certain extend by training the neural network and filling holes with “RhizoVision Explorer” (Figure S3). The more roots there are in the images, the more likely this segmentation mistake occurs. Although this is a disadvantage of the automated analysis pipeline, its main purpose is to provide reliable and consistent data for a qualitative biological analysis. The known systematic bias in the method is well predictable in contrast to the bias originating from different annotators. Consequently, the data obtained from the automated analysis pipeline are more robust and reliable, which is in advantage for further biological conclusions drawn from the data.

The consistency of the automated analysis results becomes especially visible regarding the RLD plots plotted from 2015/2016 to 2017 data (Figure 4). The decrease in 2015/2016 RLD profiles that is not monitored in the manual annotation data originated from the root senescence (Figure S4). The senescence could be better evaluated by the neural network than by the human annotator. In manual annotation, the slight, gradual discoloration of the roots visually revealing the senescence is easy to miss. Furthermore, it is a complicated work step in “Rootfly” to eliminate already annotated roots at the right point in the timeline. Taking this into consideration, the results of the method comparison for 2015/2016 and 2017 data show impressive results, regarding the accuracy and comparability of the automated analysis pipeline.

Regarding the biological conclusions that could be derived from the data, the differences between the methods are negligible, as we are working with minirhizotron data that cover a huge spatial and temporal resolution and are measured in heterogeneous conditions. Especially the consistent low Δ*RL* and NRMSE (Table 2), as well as the high conformity of the RLD profiles (Figure 4), indicate that the qualitative conclusions derived from data provided by the automated analysis pipeline and are at least the same as from manual annotation. Considering the influence of the human subjectivity on manual annotation, the automated pipeline additionally provides objectivity that most likely cannot be reached, if more than one annotator does the manual annotation.

The manual annotation itself requires a certain level of expertise in root phenotyping. This expertise is gained with a lot of personal experiences [8, 14]. Therefore, it can be hypothesized that there is also a significant influence of subjectivity in human annotation. Over the years, different persons annotated the root datasets. Hence, the impact of differences resulting from varying manual annotation strategies might influence the results more than the differences between manual and automated analysis. The direct comparison between two annotators showed a lower correlation between the persons annotating than between the automated analysis pipeline and each human annotator. Consequently, we concluded that the human effect on manual annotation is higher than the impact of a mistake done by the automated workflow.

The automated analysis pipeline provides a level of objectivity, a human annotator cannot achieve. Therefore, it is highly probable that with the application of the automated pipeline associated minimization of the human influence will significantly improve objectivity and also accuracy of the minirhizotron image analysis.

### 4.3. Speed and Efficiency

The speed is the pure amount of time the pipeline requires to analyze a certain amount of images. Efficiency is defined through the amount of time and labor needed to analyze a dataset in contrast to manual annotation. The time required to analyze root images by hand is enormous. The estimated time to analyze 100 cm^2^ of depicted soil is 1-1.5 h [17]. This is consistent with the results of other studies, needing approximately 1 h for annotating 17-38 images manually [16]. Intern evaluation reproduced the same results. To annotate 25,000 images, which is approximately the amount of images for a shorter growing season, the annotation time needed is 1,000-1,500 h. The time needed to process the same amount of images with the automated pipeline is approximately 19 h, including the training of the neural network. Without the training, the segmentation and feature extraction would only take around 6.5 h for all images. The resulting benefits in time saving are massive (Figure 5). Generally, only around 1.2%-1.9% of the time needed for manual annotation is needed by the automated workflow to process the data, including the training. Excluding the entire training process, the automated workflow requires only 0.4%-0.65% of the time needed to annotate the same amount of images manually with, e.g., “Rootfly.” Regarding the advantages of time saving, it further has to be taken into account that the time of interaction with the computer is decimated to almost zero, once the training is completed.

### 4.4. Limitations and Further Improvement

Although the current automated analysis pipeline does include time series in form of either root length density depth profiles at different time points or in form of root arrival curves, i.e., root length as a function of time at different depths, individual roots and their phenology are not followed from their birth to their death. This could be of high interest, for example, to root ecologists. To fully exploit minirhizotron data, it would be a significant progress to add a single root tracking possibility, including root order and status. The implementation of these functions would improve the pipeline and enhance the use cases for root ecologists.

## 5. Conclusion

We propose a new approach to analyze large amounts of 2D root image data. This became necessary with the big amount of data created in experimental field sites such as the minirhizotron facilities in Selhausen (Germany) as well as others [48, 49]. The automated analysis pipeline illustrated in this study is a suitable solution to easily and accurately analyze minirhizotron images in significantly less time. To the best of our knowledge, we are the first study testing a deep learning and automated feature extraction combining high-throughput minirhizotron image analysis pipeline to this extent. The biggest advantage of the automated workflow is the massive saving in time. Precisely expressed, the required time is reduced by more than 98% in contrast to manual annotation, while providing several root traits, including number of root tips, number of branch points, root length, branching frequency, network area, perimeter, volume, surface area, and diameter on a spatiotemporal scale. The required root traits can be made available quickly which may speed up further analysis and applications of this type of data. In conclusion, the automated pipeline outperforms the manual annotation in time requirements and information density, while providing reliable data and feasibility for everyone. Tested with more than 107,000 minirhizotron images, including more than 36,500 images for detailed comparison, obtained from two growing seasons and different soil types, depths and cultures our results indicate a high general validity for the presented pipeline. Irregularities in the match of manual annotation and analysis pipeline can be essentially explained with rarely occurring missed segmentations of root fragments by the automated analysis pipeline, due to soil covered roots and mainly by the influence of human subjectivity in manual annotation. Balanced training datasets and consequent annotation of the training data are the key to good results. If these facts are considered, the here presented and evaluated pipeline has the potential to be the new standard method for reliable high-throughput root phenotyping of minirhizotron images.

## Figures and Tables

**Table 1:** Overview of the camera systems and experiment timeline of minirhizotron images acquisition.

Camera system	Bartz	VSI
Original resolution (px)	754 × 510	3280 × 2464
Converted resolution (px)	1508 × 1020	2060 × 2060
Real size (mm)	16.5 × 23.5	20 × 20
Growing season	2015/2016 and 2017	2017
Culture	2015/2016: *Triticum aestivum* cv. Ambello 2017: *Zea mays* cv. Zoey	*Zea mays* cv. Zoey
Time period (dd/mm/yy)	16/11/15-23/06/16 23/06/17-12/09/17	08/06/17-22/06/17

**Table 2:** Overview of the statistical comparison of automated and manual annotation. *Δ*RL is the difference between the mean total root length (mm) obtained from automated and manual analysis methods, and a Welsch two sample *t* test shows whether differences are significant (∗*p* < 0.01).

Measurement date		2017	
		*R*_*UT*_	*R*_*LT*_
1	∆*RL*	0.45	0.42
	NRMSE	0.071	0.077
	*r*	0.92	0.53

2	∆*RL*	0.89	1.17
	NRMSE	0.071	0.053
	*r*	0.78	0.83

3	∆*RL*	0.95	0.54
	NRMSE	0.057	0.052
	*r*	0.83	0.92

4	∆*RL*	2.94^∗^	0.65
	NRMSE	0.051	0.055
	*r*	0.88	0.9

5	∆*RL*	1.36	0.92
	NRMSE	0.041	0.072
	*r*	0.9	0.87

6	∆*RL*	1.35	1.7
	NRMSE	0.044	0.065
	*r*	0.9	0.84

7	∆*RL*	-1.46	1.8
	NRMSE	0.046	0.058
	*r*	0.88	0.93

8	∆*RL*	0.11	0.55
	NRMSE	0.045	0.073
	*r*	0.86	0.89

9	∆*RL*	-1.41	-0.97
	NRMSE	0.039	0.057
	*r*	0.88	0.94

10	∆*RL*	-2.44	-2.89
	NRMSE	0.039	0.047
	*r*	0.88	0.92

11	∆*RL*		0.65
	NRMSE		0.065
	*r*		0.92

## Data Availability

(i) The supplementary data that support the findings of this study and help to operate the in this work introduced root image analysis pipeline, including an example, are open available. Furthermore, data and scripts to reproduce the RLD profiles (Figure 4) and RAC-curves (Figure S2) are open to access with the same identifier: doi:10.34731/pbn7-8g89. (ii) [29] is available at: https://github.com/Abe404/root_painter. (iii) RhizoVision Explorer [31, 40] is available at: https://zenodo.org/record/4095629 and https://github.com/rootphenomicslab/RhizoVisionExplorer

## Supplementary Materials

Supplementary Materials Figure S1: 3D spatiotemporal distribution of RLD measured in all tubes at one minirhizotron. Distances between tubes are not to scale. 1-8 represents the time steps. Figure S2: Comparison of root arrival curves of the data obtained from images originating from two minirhizotrons in the growing season 2017. The images were analyzed by hand (left: manual) and by the automated analysis pipeline (right: automated). 2017: (a) RUT manual, (b) RUT automated, (c) RLT manual, and (d) RLT automated. Figure S3: Manual vs. automated analysis. The automated analysis misses a small part of the root and underestimates the total root length slightly. Figure S4: Root senescence visible from early to late measurement dates in the growing season 2015/16 and the corresponding segmentation and skeletonization. Table S1: Detailed overview of the images taken at the growing season 2015/2016 and 2017 Table S2: Comparison of the automated analysis pipeline and the manual annotation of the total root length obtained in the growing season 2017 with a linear regression. The confidence interval (95%) of the regression coefficient (ordinary least products) are listed in parenthesis. The bias is fixed if the 95% CI of the intercept do not include 0 and the bias is proportional if the 95% CI of the slope do not include 1.
