# Supplementary material for: Development and Validation of a Deep Learning Based Automated Minirhizotron Image Analysis Pipeline
Source: Plant Phenomics. 2022 May 28;2022:9758532. doi: 10.34133/2022/9758532 (PMC9168891; doi:10.34133/2022/9758532)
Supplement: Supplementary Materials — Figure S1: 3D spatiotemporal distribution of RLD measured in all tubes at one minirhizotron. Distances between tubes are not to scale. 1-8 represents the time steps. Figure S2: Comparison of root arrival curves of the data obtained from images originating from two minirhizotrons in the growing season 2017. The images were analyzed by hand (left: manual) and by the automated analysis pipeline (right: automated). 2017: (a) RUT manual, (b) RUT automated, (c) RLT manual, and (d) RLT automated. Figure S3: Manual vs. automated analysis. The automated analysis misses a small part of the root and underestimates the total root length slightly. Figure S4: Root senescence visible from early to late measurement dates in the growing season 2015/16 and the corresponding segmentation and skeletonization. Table S1: Detailed overview of the images taken at the growing season 2015/2016 and 2017 Table S2: Comparison of the automated analysis pipeline and the manual annotation of the total root length obtained in the growing season 2017 with a linear regression. The confidence interval (95%) of the regression coefficient (ordinary least products) are listed in parenthesis. The bias is fixed if the 95% CI of the intercept do not include 0 and the bias is proportional if the 95% CI of the slope do not include 1. [file 9758532.f1.zip › 9758532.f1/Supplementary_material.pdf]

Supplementary Materials

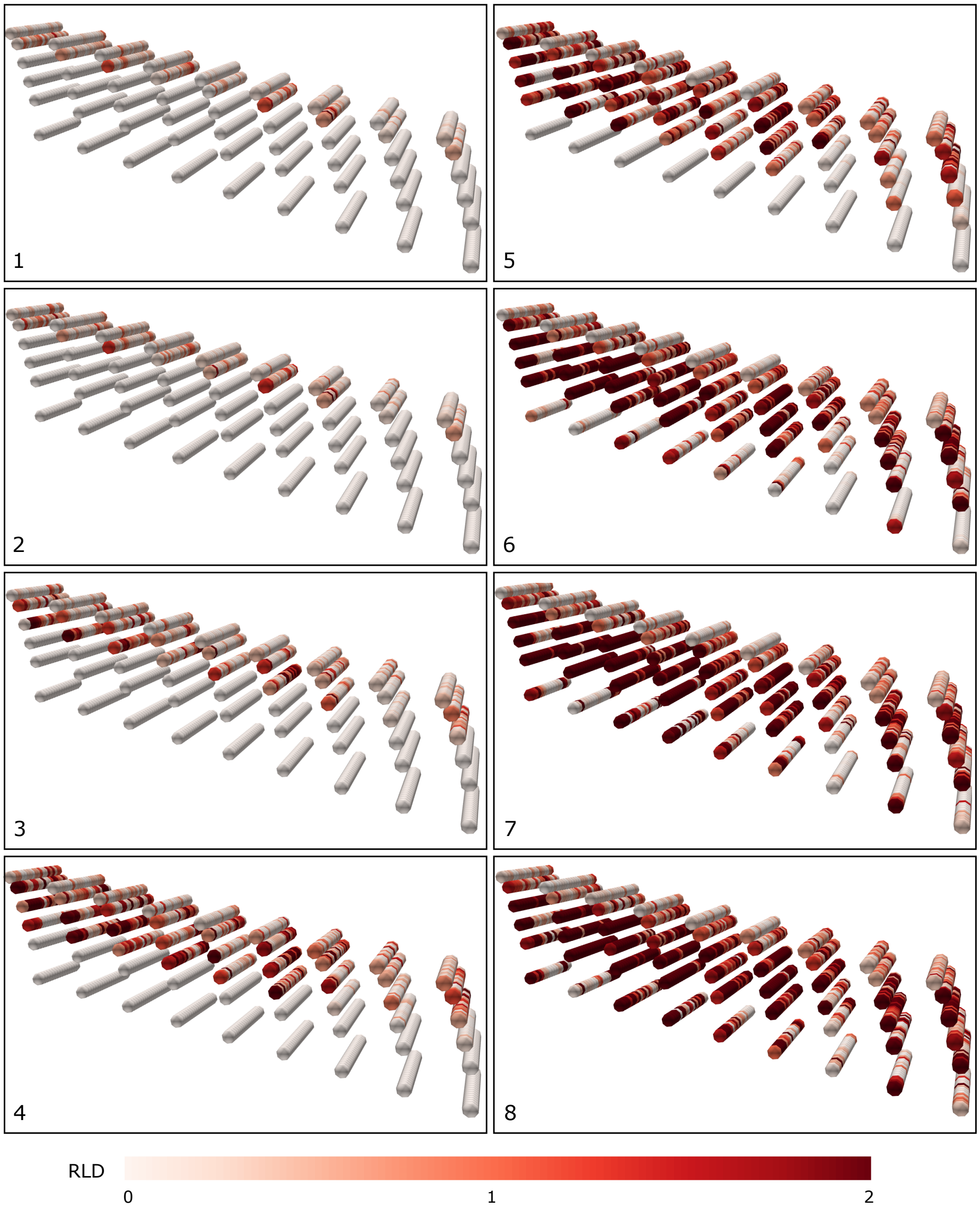

Figure S1: 3D spatio-temporal distribution of RLD measured in all tubes at one minirhizotron. Distances between tubes are not to scale. 1-8 represents the time steps.

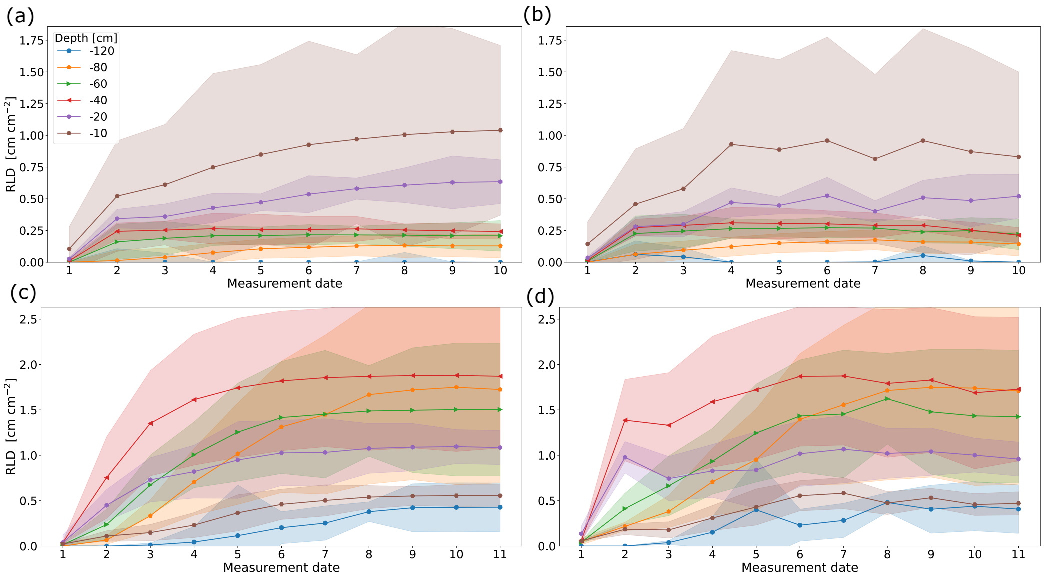

Figure S2: Comparison of root arrival curves of the data obtained from images originating from two minirhizotrons in the growing season 2017. The images were analyzed by hand (left: manual) and by the automated analysis pipeline (right: automated). 2017: a) R_{UT} manual, b) R_{UT} automated, c) R_{LT} manual,d) R_{LT} automated.

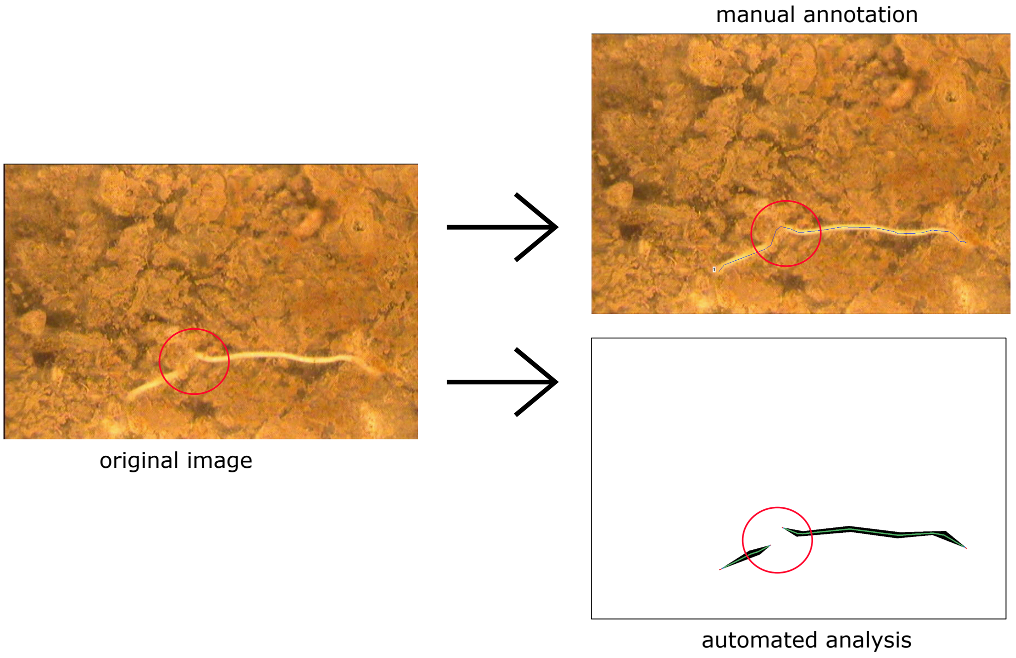

Figure S3: Manual vs. automated analysis. The automated analysis misses a small part of the root and underestimates the total root length slightly.

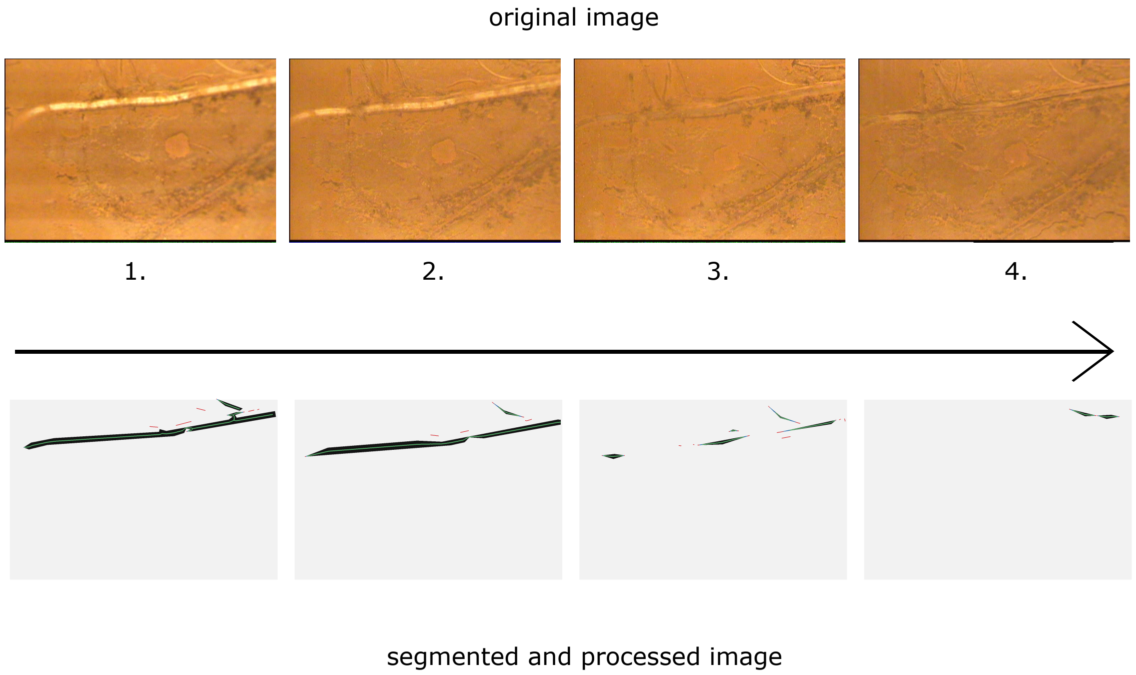

Figure S4: Root senescence visible from early to late measurement dates in the growing season 2015/16 and the corresponding segmentation and skeletonization.

Table S1: Detailed overview of the images taken at the growing season 2015/16 and 2017

2015/16				2017	
measurement no.	facility	date	images	date	images
1	R_{UT}	16/11/15	720	08/06/17	480
	R_{LT}	16/11/15	720	08/06/17	584
2	R_{UT}	26/11/15	1,080	29/06/17	1,800
	R_{LT}	26/11/15	1,079	22/06/17	1,800
3	R_{UT}	17/12/15	1,800	06/07/17	1,800
	R_{LT}	17/12/15	1,439	29/06/17	2,160
4	R_{UT}	02/02/16	1,520	13/07/17	1,800
	R_{LT}	21/01/16	1,800	06/07/17	2,160
5	R_{UT}	12/02/16	1,800	20/07/17	1,800
	R_{LT}	12/02/16	1,800	13/07/17	2,160
6	R_{UT}	26/02/16	1,800	27/07/17	1,200
	R_{LT}	26/02/16	2,160	20/07/13	2,160
7	R_{UT}	14/03/16	1,800	02/08/17	1,840
	R_{LT}	14/03/16	2,160	27/07/17	1,430
8	R_{UT}	26/03/16	1,840	10/08/17	1,959
	R_{LT}	24/03/16	2,160	02/08/17	2,159
9	R_{UT}	07/04/16	2,160	23/08/17	2,120
	R_{LT}	07/04/16	2,160	10/08/17	2,160
10	R_{UT}	13/04/16	2,160	12/09/17	1,800
	R_{LT}	13/04/16	2,160	24/08/17	2,159
11	R_{UT}	29/04/16	2,160	-	-
	R_{LT}	29/04/16	2,160	12/09/17	2,150
12	R_{UT}	06/05/16	2,160	-	-
	R_{LT}	06/05/16	2,160	-	-
13	R_{UT}	13/05/16	2,160	-	-
	R_{LT}	13/05/16	2,160	-	-
14	R_{UT}	20/05/16	2,160	-	-
	R_{LT}	20/05/16	2,160	-	-
15	R_{UT}	27/05/16	2,160	-	-
	R_{LT}	27/05/16	2,159	-	-
16	R_{UT}	03/06/16	2,160	-	-
	R_{LT}	03/06/16	2,159	-	-
17	R_{UT}	09/06/16	2,160	-	-
	R_{LT}	09/06/16	2,160	-	-
18	R_{UT}	16/06/16	2,155	-	-
	R_{LT}	16/06/16	2,160	-	-
19	R_{UT}	23/06/16	2,149	-	-
	R_{LT}	23/06/16	2,156	-	-

Table S2: Comparison of the automated analysis pipeline and the manual annotation of the total root length obtained in the growing season 2017 with a linear regression. The confidence interval (95%) of the regression coefficient (ordinary least products) are listed in parenthesis. The bias is fixed if the 95% CI of the intercept do not include 0 and the bias is proportional if the 95% CI of the slope do not include 1.

measurement no.	facility	Intercept (95% CI)	Slope (95% CI)	Bias<br>Fixed	 Proportional
1	R_{UT}	0.26 (0.08, 0.45)	0.73 (0.71, 0.74)	yes	yes
	R_{LT}	1.09 (0.83, 1.36)	0.7 (0.65, 0.75)	yes	yes
2	R_{UT}	2.8 (2.16, 3.44)	0.81 (0.78, 0.84)	yes	yes
	R_{LT}	3.5 (2.78, 4.23)	0.92 (0.89, 0.94)	yes	yes
3	R_{UT}	3.07 (2.46, 3.69)	0.8 (0.78, 0.83)	yes	yes
	R_{LT}	3.12 (2.45, 3.79)	0.88 (0.86, 0.89)	yes	yes
4	R_{UT}	2.99 (2.28, 3.7)	0.99 (0.97, 1.02)	yes	no
	R_{LT}	4.53 (3.63, 5.43)	0.86 (0.85, 0.88)	yes	yes
5	R_{UT}	2.35 (1.72, 2.97)	0.93 (0.91, 0.95)	yes	yes
	R_{LT}	6.68 (5.65, 8.07)	0.83 (0.81, 0.85)	yes	yes
6	R_{UT}	2.43 (1.75, 3.11)	0.93 (0.91, 0.95)	yes	yes
	R_{LT}	8.99 (7.51, 10.48)	0.82 (0.8, 0.84)	yes	yes
7	R_{UT}	1.55 (0.86, 2.24)	0.81 (0.79, 0.83)	yes	yes
	R_{LT}	4.99 (3.95, 6.03)	0.93 (0.91, 0.94)	yes	yes
8	R_{UT}	2.37 (1.61, 3.12)	0.85 (0.83, 0.88)	yes	yes
	R_{LT}	5.71 (4.38, 7.05)	0.89 (0.87, 0.91)	yes	yes
9	R_{UT}	1.18 (0.57, 1.79)	0.82 (0.81, 0.84)	yes	yes
	R_{LT}	3.0 (1.95, 4.05)	0.92 (0.9, 0.93)	yes	yes
10	R_{UT}	2.31 (1.65, 2.98)	0.72 (0.7, 0.74)	yes	yes
	R_{LT}	3.35 (2.2, 4.5)	0.87 (0.85, 0.88)	yes	yes
11	R_{UT}	-	-	-	-
	R_{LT}	3.61 (2.47, 4.75)	0.86 (0.84, 0.87)	yes	yes
