# Supplementary figures and images for: Development and Validation of a Deep Learning Based Automated Minirhizotron Image Analysis Pipeline
Source: Plant Phenomics. 2022 May 28;2022:9758532. doi: 10.34133/2022/9758532 (PMC9168891; doi:10.34133/2022/9758532)

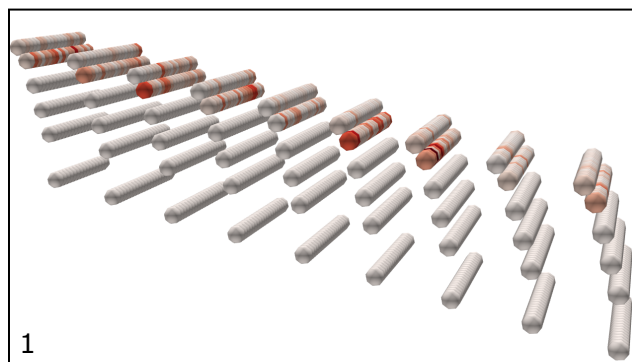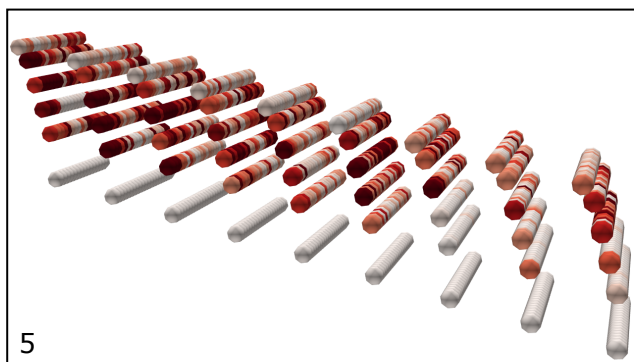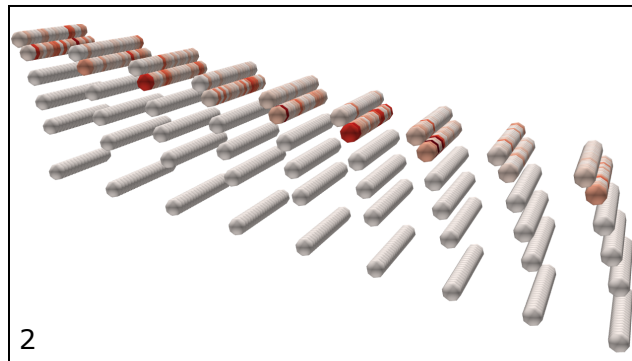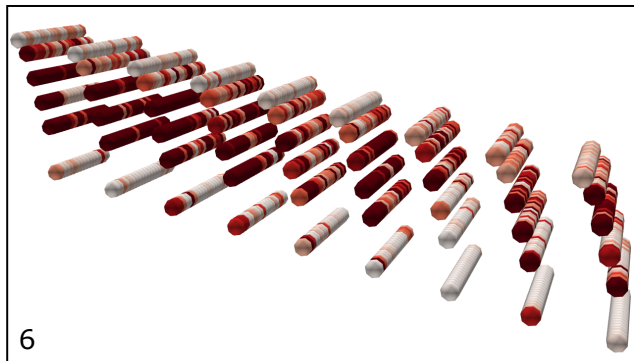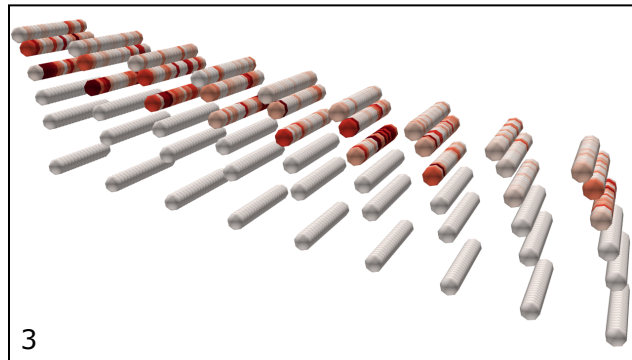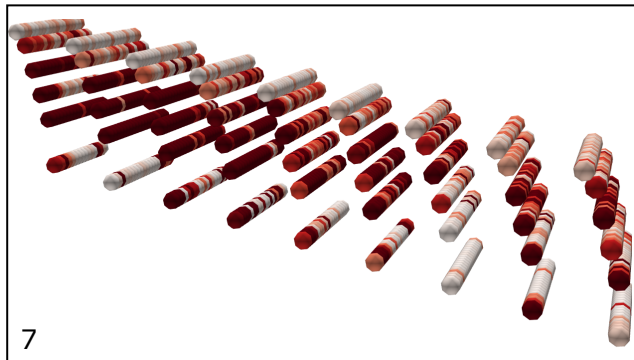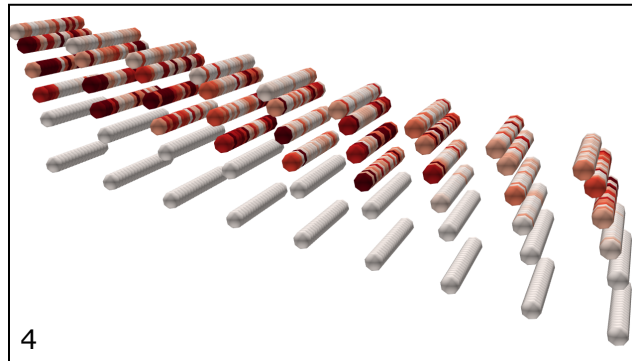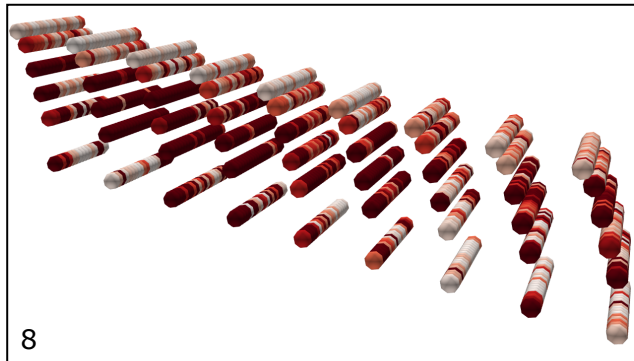

RLD

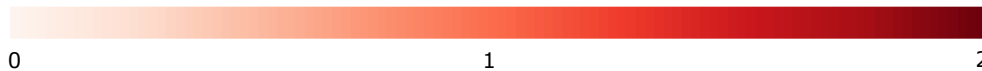

Supplement: Supplementary Materials — Figure S1: 3D spatiotemporal distribution of RLD measured in all tubes at one minirhizotron. Distances between tubes are not to scale. 1-8 represents the time steps. Figure S2: Comparison of root arrival curves of the data obtained from images originating from two minirhizotrons in the growing season 2017. The images were analyzed by hand (left: manual) and by the automated analysis pipeline (right: automated). 2017: (a) RUT manual, (b) RUT automated, (c) RLT manual, and (d) RLT automated. Figure S3: Manual vs. automated analysis. The automated analysis misses a small part of the root and underestimates the total root length slightly. Figure S4: Root senescence visible from early to late measurement dates in the growing season 2015/16 and the corresponding segmentation and skeletonization. Table S1: Detailed overview of the images taken at the growing season 2015/2016 and 2017 Table S2: Comparison of the automated analysis pipeline and the manual annotation of the total root length obtained in the growing season 2017 with a linear regression. The confidence interval (95%) of the regression coefficient (ordinary least products) are listed in parenthesis. The bias is fixed if the 95% CI of the intercept do not include 0 and the bias is proportional if the 95% CI of the slope do not include 1. [file 9758532.f1.zip › 9758532.f1/fig_S1.pdf]

(a)

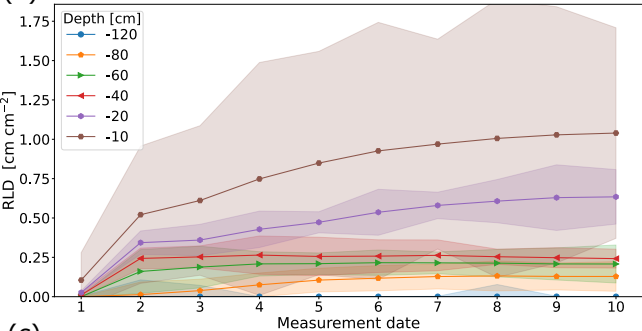

(b)

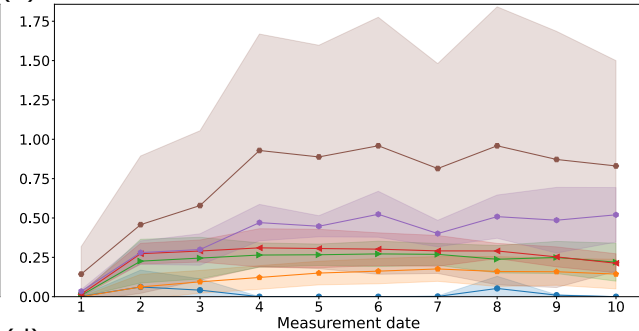

(c)

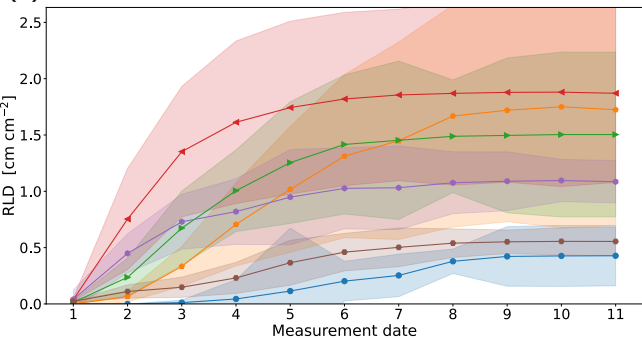

(d)

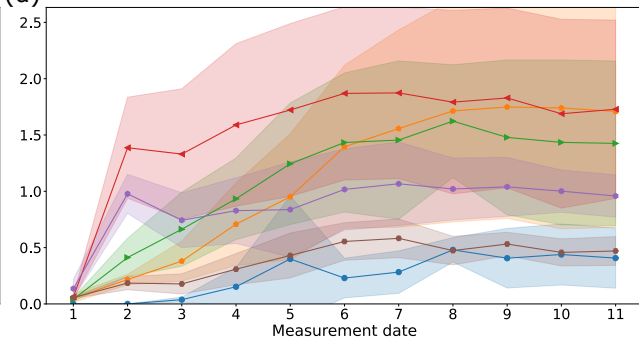

Supplement: Supplementary Materials — Figure S1: 3D spatiotemporal distribution of RLD measured in all tubes at one minirhizotron. Distances between tubes are not to scale. 1-8 represents the time steps. Figure S2: Comparison of root arrival curves of the data obtained from images originating from two minirhizotrons in the growing season 2017. The images were analyzed by hand (left: manual) and by the automated analysis pipeline (right: automated). 2017: (a) RUT manual, (b) RUT automated, (c) RLT manual, and (d) RLT automated. Figure S3: Manual vs. automated analysis. The automated analysis misses a small part of the root and underestimates the total root length slightly. Figure S4: Root senescence visible from early to late measurement dates in the growing season 2015/16 and the corresponding segmentation and skeletonization. Table S1: Detailed overview of the images taken at the growing season 2015/2016 and 2017 Table S2: Comparison of the automated analysis pipeline and the manual annotation of the total root length obtained in the growing season 2017 with a linear regression. The confidence interval (95%) of the regression coefficient (ordinary least products) are listed in parenthesis. The bias is fixed if the 95% CI of the intercept do not include 0 and the bias is proportional if the 95% CI of the slope do not include 1. [file 9758532.f1.zip › 9758532.f1/fig_S2.pdf]

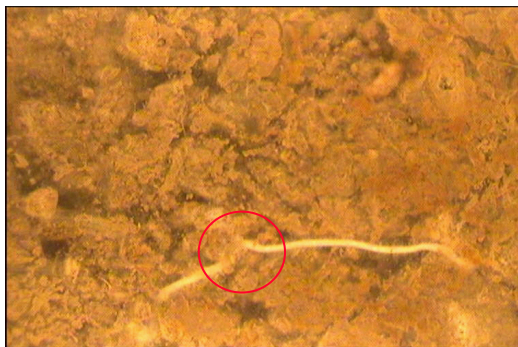

original image

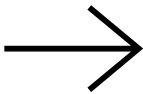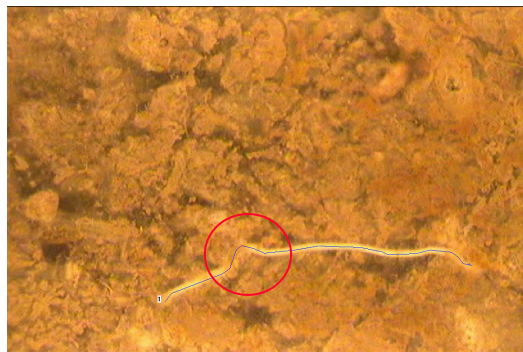

manual annotation

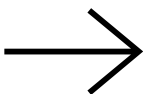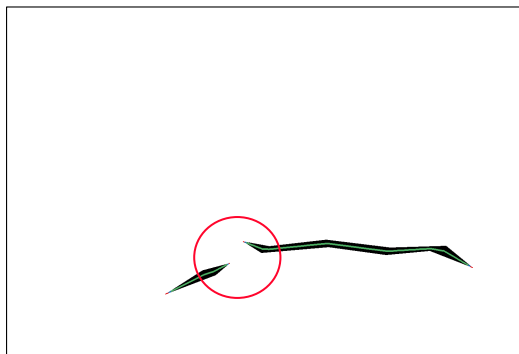

automated analysis

Supplement: Supplementary Materials — Figure S1: 3D spatiotemporal distribution of RLD measured in all tubes at one minirhizotron. Distances between tubes are not to scale. 1-8 represents the time steps. Figure S2: Comparison of root arrival curves of the data obtained from images originating from two minirhizotrons in the growing season 2017. The images were analyzed by hand (left: manual) and by the automated analysis pipeline (right: automated). 2017: (a) RUT manual, (b) RUT automated, (c) RLT manual, and (d) RLT automated. Figure S3: Manual vs. automated analysis. The automated analysis misses a small part of the root and underestimates the total root length slightly. Figure S4: Root senescence visible from early to late measurement dates in the growing season 2015/16 and the corresponding segmentation and skeletonization. Table S1: Detailed overview of the images taken at the growing season 2015/2016 and 2017 Table S2: Comparison of the automated analysis pipeline and the manual annotation of the total root length obtained in the growing season 2017 with a linear regression. The confidence interval (95%) of the regression coefficient (ordinary least products) are listed in parenthesis. The bias is fixed if the 95% CI of the intercept do not include 0 and the bias is proportional if the 95% CI of the slope do not include 1. [file 9758532.f1.zip › 9758532.f1/fig_S3.pdf]

original image

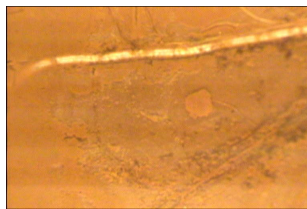

1.

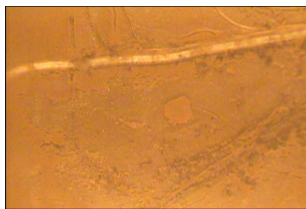

2.

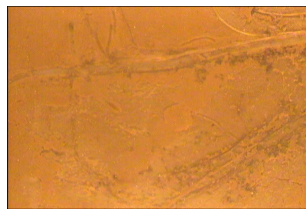

3.

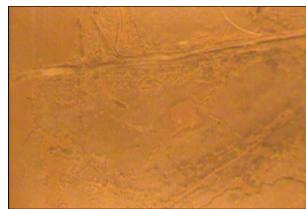

4.

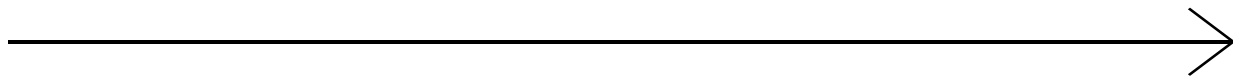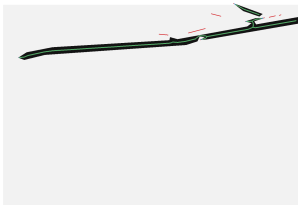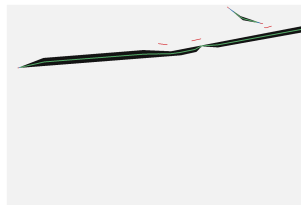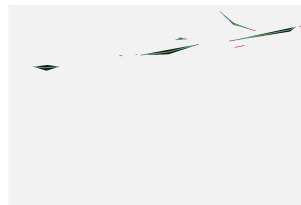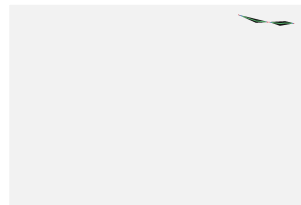

segmented and processed image

Supplement: Supplementary Materials — Figure S1: 3D spatiotemporal distribution of RLD measured in all tubes at one minirhizotron. Distances between tubes are not to scale. 1-8 represents the time steps. Figure S2: Comparison of root arrival curves of the data obtained from images originating from two minirhizotrons in the growing season 2017. The images were analyzed by hand (left: manual) and by the automated analysis pipeline (right: automated). 2017: (a) RUT manual, (b) RUT automated, (c) RLT manual, and (d) RLT automated. Figure S3: Manual vs. automated analysis. The automated analysis misses a small part of the root and underestimates the total root length slightly. Figure S4: Root senescence visible from early to late measurement dates in the growing season 2015/16 and the corresponding segmentation and skeletonization. Table S1: Detailed overview of the images taken at the growing season 2015/2016 and 2017 Table S2: Comparison of the automated analysis pipeline and the manual annotation of the total root length obtained in the growing season 2017 with a linear regression. The confidence interval (95%) of the regression coefficient (ordinary least products) are listed in parenthesis. The bias is fixed if the 95% CI of the intercept do not include 0 and the bias is proportional if the 95% CI of the slope do not include 1. [file 9758532.f1.zip › 9758532.f1/fig_S4.pdf]
